# Differential Extracellular Matrix Production by Mesenchymal Stromal Cells From Foetal Skin, Adult Skin and Burn Eschar

**DOI:** 10.1111/wrr.70202

**Published:** 2026-10-06

**Authors:** Madalena L. N. P. Gomes, Marcel Vlig, Amran Dinle, Paul A. J. Krijnen, Esther Middelkoop, Hans W. M. Niessen, Bouke K. H. L. Boekema

**Affiliations:** ^1^ Department of Pathology Amsterdam University Medical Center (AUMC) Amsterdam the Netherlands; ^2^ Department of Plastic, Reconstructive and Hand Surgery AUMC, Location VUmc Amsterdam the Netherlands; ^3^ Tissue Function and Regeneration Amsterdam Movement Sciences Research Institute Amsterdam the Netherlands; ^4^ Burn Research Lab Alliance of Dutch Burn Care Beverwijk the Netherlands; ^5^ Department of Biomedical Laboratory Research Hogeschool Leiden Leiden the Netherlands; ^6^ Amsterdam Cardiovascular Sciences Research Institute, AUMC, Location VUmc Amsterdam the Netherlands; ^7^ Alliance of Dutch Burn Care, Burn Center Red Cross Hospital Beverwijk the Netherlands; ^8^ Department of Cardiac Surgery AUMC, Location VUmc Amsterdam the Netherlands

**Keywords:** collagen, proteoglycan, regeneration, vitamin C, wound healing

## Abstract

After full‐thickness injuries, foetal skin regenerates, while adult skin heals with scarring. Upon injury, mesenchymal stromal cells (MSCs) produce extracellular matrix (ECM) that shapes the wound microenvironment by providing structure, regulating the availability of soluble factors and directing cell behaviour. Therefore, the ECM secreted by MSCs likely guides the healing outcome towards either scarless or scar prone healing. We analysed the ECM produced by primary human MSCs from foetal skin (hfS‐MSCs; regenerative/healthy), adult dermis (hD‐MSCs; scarring/healthy) and burn eschar tissue (hBE‐MSCs; scarring/wounded). We first optimised ECM production across MSC populations, then quantified total ECM deposition, collagen and total protein content and analysed ECM‐related genes. Finally, we assessed collagen maturity in foetal versus adult skin. Standard medium containing 10 μg/mL of magnesium ascorbyl phosphate stimulated ECM deposition in all MSC populations, while minimising ECM area reduction and contraction. Total protein, collagen gene and protein expressions were higher in hfS‐MSC‐derived ECM, while ECMs derived from hBE‐MSCs and hD‐MSCs showed an increased proportion of non‐collagenous proteins. Collagen I:V gene ratio was decreased in hfS‐MSCs, while collagen I:III gene ratio was increased in hBE‐MSCs compared to hD‐MSCs. Both hfS‐MSCs and hBE‐MSCs showed increased ECM deposition and collagen insolubility. Moreover, hBE‐MSCs showed higher ECM contraction, as well as PLOD2 and fibronectin gene expression. Foetal skin exhibited higher immature collagen, whereas adult skin exhibited higher mature collagen. We revealed intrinsic differences in ECM amount, composition and ECM‐related gene expression between MSC populations and their tissues of origin, suggesting potential ECM‐related biomarkers underlying regeneration or repair.

**Abbreviations**
BCA: bicinchoninic acid
ECM: extracellular matrix
ISCT: International Society for Cell and Gene Therapy
LH: lysyl hydroxylase
MFI: mean fluorescence intensity
MSC: mesenchymal stromal cell
PBS: phosphate‐buffered saline
VITC: magnesium ascorbyl phosphate

## 1 Introduction

Full‐thickness burns, especially those involving extensive total body surface area, cause lifelong scarring that can impair function and comfort, thereby reducing quality of life [1, 2]. Unlike full‐thickness wounds in adults (and children), foetal skin wounds in early development regenerate and do not result in scar formation [3]. Upon injury, mesenchymal stromal cells (MSCs)—a heterogeneous population that includes fibroblasts, myofibroblasts and stem/progenitor cells [4]—generate new tissue by secreting extracellular matrix (ECM) proteins, which store/release soluble factors and serve as a scaffold, directing cell phenotype and function [5]. Therefore, the ECM plays an essential role in shaping the wound microenvironment [6].

Collagens, proteoglycans, glycosaminoglycans and core glycoproteins—fibronectin and elastin—are vital ECM components [7, 8]. Types I, III and V are the predominant types of collagen in skin [7], with collagen I being most abundant in both foetal and adult skin. During prenatal development and aging, the proportion of collagens III and V decreases, resulting in lower levels in adult skin [3]. Human foetal fibroblasts show increased gene expression of collagens I and V compared to adults and collagen deposition begins earlier in foetal wounds compared to adult wounds [3]. Versican, decorin and biglycan are the most expressed proteoglycans in skin, influencing cell proliferation, migration and phenotype [9, 10]. Foetal rat fibroblasts exhibit reduced decorin gene expression compared to adult fibroblasts [3]. Moreover, decorin is also decreased in hypertrophic scar tissue compared to normal skin [11]. Hyaluronic acid, a glycosaminoglycan, is differentially expressed in foetal versus adult skin and wounds in rabbits [3]. Fibronectin is involved in the organisation of the granulation tissue, promoting the spreading of platelets, as well as the adhesion and migration of immune, mesenchymal, endothelial and epidermal cells in the wounded area [3, 12]. Expression of fibronectin is upregulated in human foetal skin and deposition of fibronectin starts earlier in rabbit foetal wounds [3]. Finally, lysyl hydroxylase‐2 (LH‐2), encoded by PLOD2 and crucial for collagen crosslinking, collagen solubility and fibrillogenesis [13], is also involved in fibrosis [14]. Human foetal fibroblasts exhibit higher baseline expression of LH, but under in vitro wound conditions, PLOD2 expression increases only in adult fibroblasts [3].

Previously, we reported that nearly 70% of ECM‐related proteins exhibited inconsistent expression patterns across studies using models from different species [3]. In healthy conditions, four of the 10 above mentioned targets were either studied exclusively in animal models or not studied at all; under wound conditions, all 10 targets were investigated solely using animal models or remained unstudied. Furthermore, conditions were used that did not stimulate ECM production. Studies on ECM production by human skin fibroblasts in vitro have focused on neonatal/adult fibroblasts, not on foetal or eschar MSCs [15, 16, 17, 18, 19]. Additionally, collagen deposition varies with fibroblast origin. Keloid fibroblasts, for example, show a higher response to vitamin C than adult skin fibroblasts [19]. Hence, optimising culture conditions was crucial before studying ECM production across MSC populations.

We aimed to explore the differences between ECM derived from cells isolated from tissue with regenerative capacity (foetal skin) and ECM derived from tissue that undergoes reparative healing associated with scar formation (adult skin and wounds). To do so, we stimulated ECM secretion in healthy human foetal, adult and wounded (burn eschar) skin MSCs for 14 days. We compared ECM production levels and differential ECM‐related gene expression. This study comprises two main parts: optimising ECM production by adjusting culture conditions, particularly the magnesium ascorbyl phosphate (VITC) concentration; and examining ECM differences across MSC populations.

By investigating the differences in ECM production rate and composition, we may identify potential regenerative or fibrosis biomarkers, ultimately contributing to advance the treatment of burn wounds and improve the quality of life of these patients.

## 2 Materials and Methods

### 2.1 Human Tissue Collection & Ethical Considerations

Healthy skin samples and eschar tissue were obtained from patients undergoing elective abdominoplasty or burn treatment, respectively, via the Residual Tissue Biobank of the Red Cross Hospital, Beverwijk, The Netherlands, between 2004 and 2024. The Medical Ethics Review Committee determined that the research was exempt from the Medical Research Involving Human Subjects Act. The use of these coded, post‐operative residual tissue samples was approved by patients through the informed opt‐out‐plus protocol of the Red Cross Hospital (https://www.coreon.org/wp‐content/uploads/2023/06/Code‐of‐Conduct‐for‐Health‐Research‐2022.pdf). Foetal skin tissue, collected between 2008 and 2012, was harvested during abortion procedures performed on pregnant women at the Center for Contraception, Abortion and Sexuality (CASA, Leiden and The Hague, The Netherlands). Patients were informed that they could withdraw without providing a reason. Foetal skin tissue, collected in 2023, was obtained from the HIS Mouse Facility of the Academic Medical Center, Amsterdam. All material has been collected from donors from whom written informed consent for the use of the material for research purposes had been obtained by the Bloemenhove clinic (Heemstede, The Netherlands). These informed consents are kept together with the medical record of the donor by the clinic. The use of foetal tissues used in this study complies with Dutch law (*Wet foetaal weefsel*), which permits foetal material to be utilised exclusively for medical purposes, scientific research and education. General donor information is provided in Table 1 and in Section 2.9.

**TABLE 1:** Information of MSCs and their respective donor.

Cells	Aim	Cell passage	Donor age	Donor sex	Donor PBD
		Min–Max, mean ± SD	Min–Max, mean ± SD	F/M/U (%)	Min–Max, mean ± SD
hfS‐MSCs	Characterisation	1–3, 1.8 ± 0.8	15–22, 18 ± 3.0 gw	80/20/0	n.a.
	ECM production	1–3, 1.9 ± 0.6	15–21, 18 ± 2.3 gw	13/25/62	n.a.
hBE‐MSCs	Characterisation	2–3, 2.4 ± 0.5	37–64, 53 ± 11 years	60/40/0	11–19, 15 ± 2.9
	ECM production	1–3, 2.1 ± 0.6	9–69, 28 ± 21 years^a^	75/25/0	12–30, 20 ± 6.8, U = 1
hD‐MSCs	Characterisation	2–4, 3.4 ± 0.9	28–76, 54 ± 18 years	40/60/0	n.a.
	ECM production	1–4, 2.4 ± 1.1	23–49, 37 ± 11 years, U = 2	88/12/0	n.a.

Abbreviations: F, female; gw, gestational week; M, male; n.a., not applicable; PBD, post‐burn day; U, unknown.

^a^ One child (< 12 years) and 4 adolescents (14–19 years).

### 2.2 Isolation of Primary Human Mesenchymal Stromal Cells

Split‐skin grafts from healthy adult skin, burn wound eschar tissue and foetal skin were processed using enzymatic digestion, followed by filtration and centrifugation to isolate cells, as detailed in Supporting Information. Cells were cultured in standard medium (DMEM supplemented with 10% foetal calf serum, 1% penicillin/streptomycin and 1% GlutaMAX), subsequently trypsinized and cryopreserved in liquid nitrogen until use. Passage numbers are provided in Table 1. The sex of foetal donors was determined as detailed in Supporting Information.

### 2.3 Characterisation of Primary Human Mesenchymal Stromal Cells

Isolated cells (5 donors/cell type) were cultured to near confluency in standard medium, then trypsinized and washed in complete FACS buffer (0.5% BSA, 0.05% sodium azide and 1 mM EDTA in PBS). Cell suspensions of 250,000 cells/donor were stained with different antibody panels (see Table S1) and were analysed by flow cytometry (MACS Quant Analyser 10, Miltenyi Biotec GmbH, Bergisch Gladbach, NW, Germany) (as detailed in Figure S1). The MSC panel was based on the International Society for Cell and Gene Therapy (ISCT) revised criteria [4]: expression of CD90, CD73 and CD105 and absence of haematopoietic markers CD14, CD19 and CD45. To exclude endothelial cell contamination, CD31 was used as a negative endothelial marker. Considering the tissue sources examined in this study and prior reports linking CD34 to distinct cellular properties in MSCs [20], CD34 was included as an additional characterisation marker.

### 2.4 Cell Culture and ECM Production

Cells were cultured for 21 days (1 donor/cell type) in six‐transwell plates at 200,000 cells/well in standard conditions (FBM) and ECM production conditions [CnT‐PR‐ECM (CellnTec, Bern, BE, Switzerland) or FBM with 65 μg/mL magnesium ascorbyl phosphate (VITC) (Sigma‐Aldrich, St. Louis, MO, USA), a more potent and stable collagen‐inducing derivative of vitamin C [16]]. Cells were also cultured in 24‐well plates at 80,000 cells/well in FBM until confluency, then treated with 0, 10, 65 or 180 μg/mL VITC for 48 h (for RNA sample collection, 4 donors/cell type) and 14 days (for protein sample collection, 3 donors/cell type). Medium was refreshed every 48 h.

### 2.5 Quantification of Gene Expression

RNA was isolated from samples after 48 h of VITC treatment, cDNA was synthesised and reverse transcriptase quantitative PCR performed, as detailed in Supporting Information. Primer pairs are detailed in Table S2. The 48‐h time point was selected based on results (data not shown) showing a similar trend in ECM expression at 48 h and 14 days, with higher expression at 48 h for key targets like collagens, ACTA2 and crosslinking‐related genes. Results were normalised to the geometric mean of YWHAZ and GPI reference genes. To reduce donor variability and highlight condition‐specific differences, *z*‐score normalisation was applied per donor across all conditions.

### 2.6 ECM Deposition

Eosin staining was used to quantify ECM deposition on Days 0, 4, 7, 9 and 14, as it stains all ECM proteins [21]. Wells were washed with phosphate‐buffered saline (PBS), incubated with 0.2% eosin at room temperature for 1 min (200 μL/well), followed by five washes with PBS. Images of each well were captured using a digital camera and analysed using ImageJ. For quantitative image‐based analyses, one well per donor–condition combination was used. Deposited ECM was estimated from mean grey value measurements within manually delineated ECM‐positive regions of interest (ROIs) within each well, serving as a proxy for staining intensity.

### 2.7 ECM Contraction

ECM contraction was assessed by two complementary parameters: ECM area contraction and frequency of ECM contraction.

1. *ECM area contraction* **—**ECM area was quantified using the same ROIs of ECM‐positive regions within each well (obtained after eosin staining and image analysis as detailed above). The extent of ECM area contraction was calculated according to the formula: ECM area contraction (%) = 100 × (1 − ECM area at Day x/Total well area), where Total well area represents the maximum ECM coverage at baseline (see methodological workflow in Figure 3A). Higher values indicate greater ECM contraction.
2. *Frequency of ECM contraction*—Only full ECM contraction events were considered for frequency analysis. Full ECM contraction was assessed daily from Days 0 to 14 and defined by a distinct macroscopic observation, characterised by condensation of ECM into a compact, dot‐like structure within the well. This endpoint corresponds to near‐complete loss of ECM coverage and was previously validated as representing ~100% ECM area contraction based on eosin‐stained image analysis of representative samples. For frequency analysis, 10 wells per donor–condition combination were used. The frequency of contraction was calculated as: Frequency of ECM contraction (%) = 100 × Number of wells exhibiting ECM contraction/Total number of wells (see methodological workflow in Figure 3A).

### 2.8 Total Protein and Collagen Quantification

At Days 4, 9 and 14, ECM and cells were digested with pepsin in acidic solution and collected for analysis. Following overnight incubation, samples were centrifuged to separate soluble and insoluble fractions. Soluble and insoluble collagen contents were quantified using SIRCOL assays (Biocolor, Carrickfergus, NIR, UK) and total protein in the soluble fraction was measured by Pierce Bicinchoninic Acid (BCA) Protein Assay (Thermo Scientific, Waltham, MA, USA) assay. Detailed procedures and assay adaptations are provided in Supporting Information.

### 2.9 Histology

Foetal and adult skin samples were obtained from six donors per tissue type. Foetal donors ranged from 16 to 22 gestational weeks (mean 19 ± 2.5 weeks), with 33% female, 33% male and 33% of unknown sex. Adult donors ranged from 26 to 65 years of age (mean 43 ± 15 years, 1 donor of unknown age), with 50% female, 33% male and 17% of unknown sex. Of these donors, two per tissue type were included in the MSC characterisation and ECM production studies.

Tissue samples were fixed with kryofix (50% ethanol, 7% PEG300) and imbedded in paraffin. Serial tissue sections (5 μm) were mounted on silane‐coated slides. To discriminate between mature collagen and granulation tissue, a Herovici staining was performed on the paraffin‐embedded sections as described previously [22]. In short, after dewaxing and rehydration, slides were incubated for 10 min in Weigert's haematoxylin solution and rinsed for 10 min in running tap water. Next, slides were incubated for 2 min in Herovici working solution, consisting of 1% Acid Fuchsin in saturated Picric acid solution mixed with 0.05% Analine Blue, 16.5% glycerol and 0.8% saturated lithium carbonate (all from Merck KGaA, Darmstadt, HE, Germany) in distilled water at a ratio of 45/55 (v/v). This was followed by 2 min decolorisation in 1% acetic acid (Fisher Scientific, Waltham, MA, USA).

### 2.10 Histological Image Analysis and Quantification

Slides were scanned at ×10 magnification using a ZEN 2 imager (Zeiss, Badhoevedorp, NH, The Netherlands) and analysed using ZEN Intellis image analysis software (ZEN 3.13, Zeiss). Images were segmented based on the distinctive colour difference between mature (purple/red) and immature (blue) stained collagen [23]. Non‐collagenous structures, including epidermis, vessels and hair follicles, were excluded from analysis through segmentation. Collagen areas were quantified within the total tissue region of interest and results were expressed as the proportion of mature and immature collagen relative to total analysed tissue area. Image‐derived measurements were exported and further processed in R (version 4.5.2) and RStudio (2026.04.0526+) [24], using packages readxl (version 1.4.5) [25] and tidyverse [26].

### 2.11 Statistical Analysis

Data is presented as mean ± standard deviation. Statistical analysis was performed in GraphPad Prism 10.2.0 (GraphPad Software Inc., Boston, MA, USA). Normality was assessed using the Shapiro–Wilk test. Parametric tests (unpaired *t*‐test for two groups; one‐way or two‐way ANOVA with Dunnett's or Fisher's least significant difference post hoc for multiple groups; one sample *t*‐test for one group) were applied to normally distributed data. Non‐parametric tests (Mann–Whitney for two groups; Kruskal–Wallis with Dunn's post hoc for multiple groups) were used otherwise. The specific statistical tests applied for each analysis are indicated in the corresponding figure legends.

## 3 Results

### 3.1 Characterisation of MSCs

Isolated cells were characterised by flow cytometry to confirm their mesenchymal origin and to assess whether they meet the criteria established by the ISCT for classification as human MSCs. Information on isolated cells and their respective donors is depicted in Table 1. Cells were plastic‐adherent under standard culture conditions (Figure 1C). All populations showed strong expression of CD90 and CD73 and moderate expression of CD105 (Figure 1A,B). Cells were negative for CD14, CD19, CD31 and CD45 (Figure 1A,B). These findings confirm that the cells used in this study are MSCs. According to the revised criteria for MSCs, the tissue of origin should be indicated in cell annotation [4]. Accordingly, we designated our cells as human adult dermis‐derived MSCs (hD‐MSCs), human foetal skin‐derived MSCs (hfS‐MSCs) and human burn eschar‐derived MSCs (hBE‐MSCs). All MSC populations showed moderate CD34 expression, with ~74% of cells positive for CD34 but in general with low MFI; notably, MFI was higher in hfS‐MSCs (Figure 1A,B).

**FIGURE 1:**
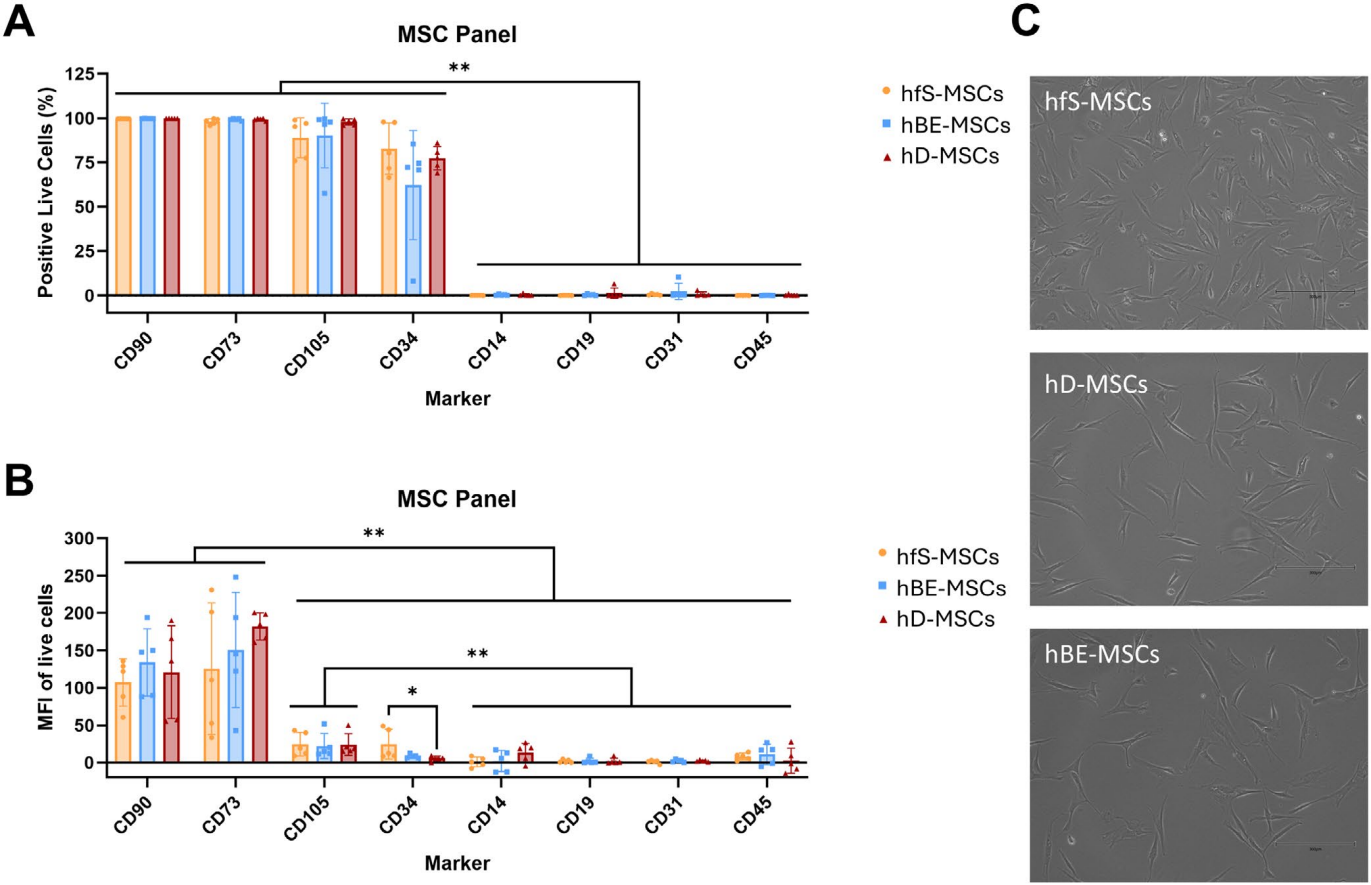
Characterisation of hfS‐MSCs, hD‐MSCs and hBE‐MSCs. Cells were isolated, cultured until Passages 1–4 and analysed by flow cytometry using a panel of CD markers. (A) Percentage of live cells positive for each marker. (B) Mean fluorescence intensity (MFI) of live cells positive for each marker. (A, B) Donors = 5/MSC type. Marker comparisons within each cell type and differences between cell types for each marker were assessed using the Kruskal–Wallis test followed by Dunn's post hoc test. *p < 0.05, **p < 0.01. (C) hfS‐MSCs, hD‐MSCs and hBE‐MSCs in standard culture conditions (one donor/MSC type shown as an example).

### 3.2 Optimisation of ECM Production Conditions

We cultured three MSC populations (information on MSCs and their respective donors is depicted in Table 1) for 21 days in standard medium (control) and two ECM‐stimulating media (CNT‐PR‐ECM and standard medium with VITC) to assess which medium yielded the highest ECM production. In standard medium conditions, none of the MSCs produced visible ECM. In CnT‐PR‐ECM or standard medium with VITC, hfS‐MSCs and hBE‐MSCs had formed ECM by Day 21 (Figure S2), while hD‐MSCs only produced ECM in standard medium with VITC. hBE‐MSC‐derived ECM contracted after 14 days, while ECM derived from hfS‐MSCs and hD‐MSCs retained their shape throughout 21 days (Figure S2). Subsequent experiments in standard medium with VITC were terminated at Day 14, when all MSCs had produced ECM, in order to prevent contraction.

#### 3.2.1 ECM‐Related Protein and Gene Expression

To determine optimal VITC concentration for ECM production by MSCs, we tested three different VITC concentrations for 14 days on MSCs in 24‐well plates. Eosin staining was used to assess ECM formation macroscopically on Days 4, 7, 9 and 14. All VITC treatments increased ECM production compared to controls based on eosin staining (Figure 2A,B). BCA assay showed also a significant increase in total protein amount on Day 14 in all VITC‐treated MSC populations compared to controls (Figure 2C–E), except for hfS‐MSCs treated with 180 μg/mL. However, no differences in eosin staining and total protein amount were found between the different VITC concentrations for all MSC types (Figure 2A–E). Collagen, a major ECM component, was quantified and only detected in VITC‐supplemented conditions, confirming VITC is needed for collagen production (Figure 2F–H). Exceptionally, hfS‐MSCs from one foetal donor produced a low amount of collagen (4.23 μg) in standard medium without VITC. We hypothesised that increased VITC concentrations could facilitate collagen crosslinking, thereby increasing collagen insolubility. Nevertheless, significant differences in the ratio insoluble/soluble collagen were not observed between VITC concentrations for all MSCs (Figure S3).

**FIGURE 2:**
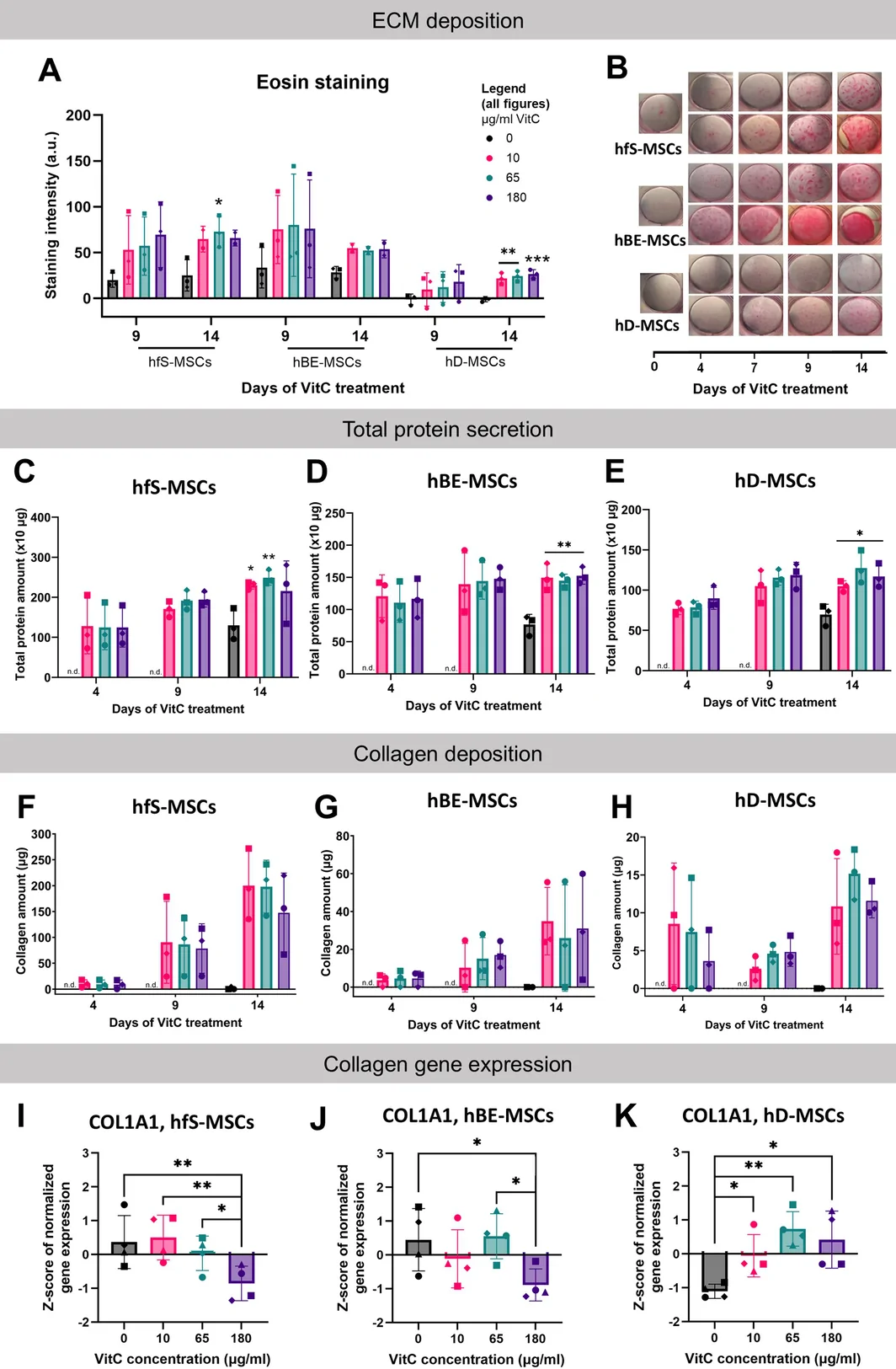
ECM‐related protein and gene expression in hfS‐MSCs, hD‐MSCs and hBE‐MSCs treated with 0 (control), 10, 65 or 180 μg/mL VITC. One point/donor, each represented by a unique symbol, 3–4 donors/MSC type. (A) Eosin staining intensity at Days 9 and 14, corrected by subtracting Day 0 value. (B) Wells stained with eosin over time, 1 donor/MSC type: Control (top row), 10 μg/mL VITC (bottom row). (C–E) Total protein (BCA assay) and (F–H) collagen (SIRCOL assay) amounts over time. (C–H) Please note that different ranges on the *y*‐axis are depicted. 1 well/donor, three technical replicates. n.d.: not determined, control measured only at the last time point due to ECM absence. (I–K) Normalised COL1A1 gene expression at 48 h in MSCs. The average of 5 wells/donor is shown. (A–K) VITC concentration comparisons were performed using one‐way ANOVA with Tukey's post hoc test or Kruskal–Wallis test with Dunn's post hoc test; however, in (F–H), statistical tests were conducted among the 10, 65 and 180 μg/mL VITC groups only and not against the control (0 μg/mL) due to zero values in the control group. *p < 0.05, **p < 0.01, ***p < 0.001, compared to control for (A–H).

COL1A1 gene expression in MSCs stimulated with different VITC concentrations was measured at 48 h. hD‐MSCs treated with VITC, irrespective of concentration, revealed a significant increase in COL1A1 expression compared to control (no VITC), while no increase was observed in hfS‐MSCs and hBE‐MSCs (Figure 2I–K). COL1A1 gene expression in hfS‐MSCs even decreased with higher VITC concentrations and both hfS‐MSCs and hBE‐MSCs treated with 180 μg/mL VITC showed significantly lower expression compared to other concentrations (Figure 2I,J). Conversely, hD‐MSCs showed no differences between 10, 65 and 180 μg/mL VITC (Figure 2K).

From these experiments, we concluded that 10 μg/mL VITC is the optimal concentration for ECM production in our experimental setting.

#### 3.2.2 ECM Contraction

hfS‐MSCs stimulated with 180 μg/mL of VITC showed higher ECM area contraction and significantly increased frequency of ECM contraction compared to 10 or 65 μg/mL (Figure 3A,C). hBE‐MSCs exhibited an increased ECM area contraction at Days 9 and 14 and increased frequency of ECM contraction, particularly, at Days 7 and 8, when stimulated with 65 or 180 μg/mL compared to 10 μg/mL (Figure 3B,D). Higher VITC concentrations may increase α‐smooth muscle actin expression [27], encoded by the ACTA2 gene, a myofibroblast marker involved in wound contraction, possibly promoting ECM contraction. However, ACTA2 gene expression showed no significant differences across VITC concentrations at 48 h (Figure 3C–E).

**FIGURE 3:**
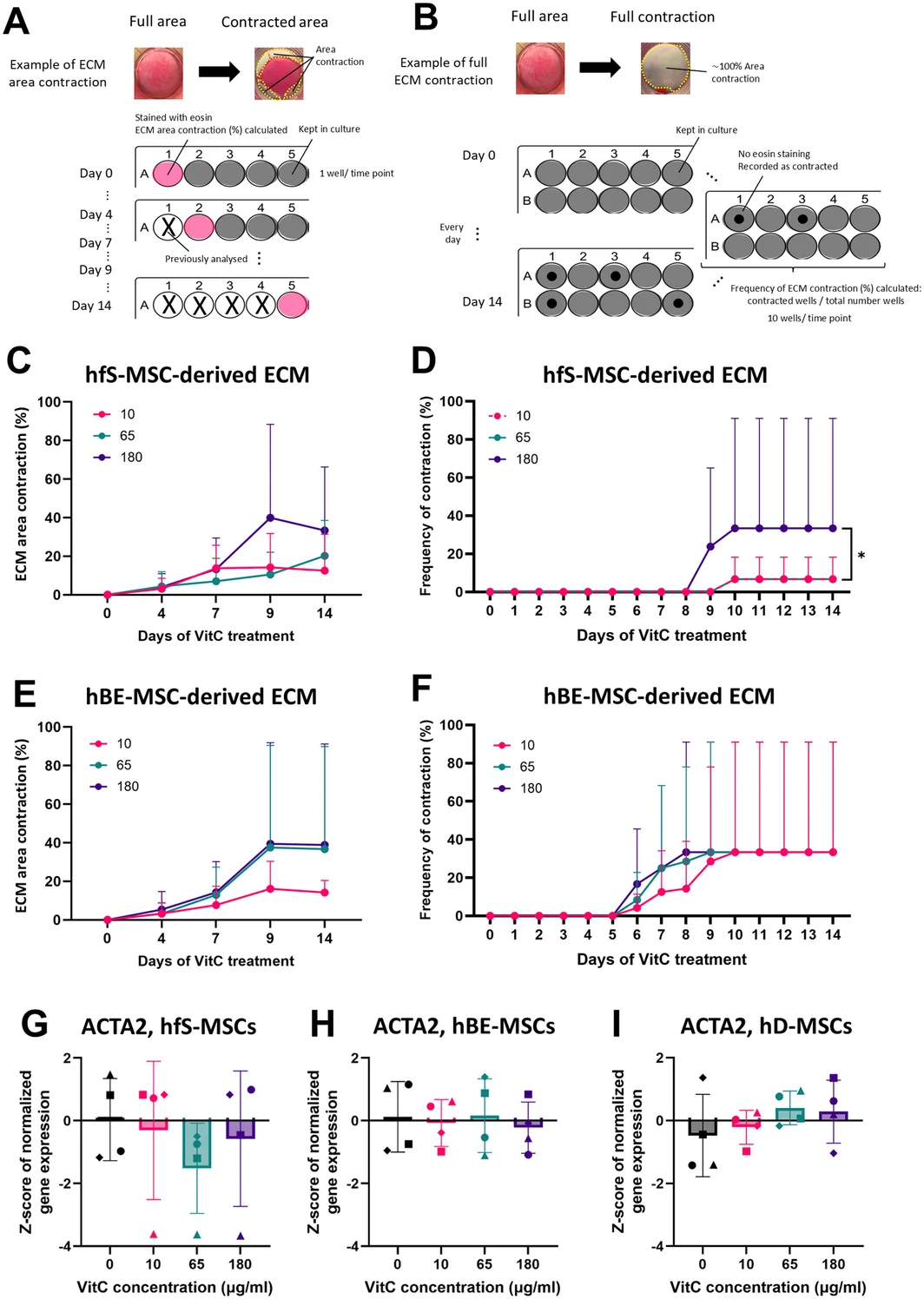
ECM area contraction and frequency of ECM contraction across VITC concentrations. (A) Definition of ECM area contraction, with example and method's explanation. (B) Definition of frequency of ECM contraction, with example and method's explanation. (C, E) Percentage of hfS‐MSC‐derived and hBE‐MSC‐derived ECM area contraction from Days 0 to 14 across 10, 65 and 180 μg/mL of VITC. One point is the average of three donors, 1 well/donor. ECM area contraction was not observed for hD‐MSC‐derived ECM. (D, F) Frequency of contraction—percentage of wells where full ECM contraction occurred (~100% ECM area contraction)—from Days 0 to 14 across 10, 65 and 180 μg/mL of VITC. One point is the average of three donors, 10 wells/donor were used. Full ECM contraction did not occur for hD‐MSC‐derived ECM. (C–F) Two‐way ANOVA followed by Tukey's multiple comparisons test was performed to assess the main effect of VITC concentration across all time points. *p ≤ 0.05. (G–I) Normalised ACTA2 gene expression at 48 h in MSCs. One point/donor, each represented by a unique symbol, 4 donors/MSC type, average of 5 wells/donor. VITC concentration comparisons were performed using one‐way ANOVA with Tukey's post hoc test or Kruskal–Wallis test with Dunn's post hoc test; no significant differences were found.

### 3.3 Differences Between ECMs From Diverse MSC Populations

#### 3.3.1 Protein Expression

To characterise the ECM produced by the different MSC populations we stimulated MSCs with 10 μg/mL VITC, the optimal concentration for ECM production determined in the earlier experiments. While previous studies have mainly focused on collagen and, to a lesser extent, glycosaminoglycans [15, 16], we used eosin staining to assess total ECM deposition more comprehensively. hD‐MSCs presented a significant three‐fold lower ECM deposition over 14 days of VITC treatment compared to hBE‐MSCs and hfS‐MSCs (Figure 4A,B). This trend of reduced protein staining in hD‐MSCs was already evident even without VITC supplementation (Figure 4A). At Day 14 of VITC treatment, total protein measured by BCA was significantly higher in hfS‐MSCs than in the other MSC populations (Figure 4C). When stimulated with VITC, hBE‐MSCs showed significantly higher total protein levels compared to hD‐MSCs (Figure 4C). Furthermore, our results showed that the amount of protein significantly increased in all MSC populations when stimulated for ECM production (with VITC) compared to standard medium conditions (Figure 4C).

**FIGURE 4:**
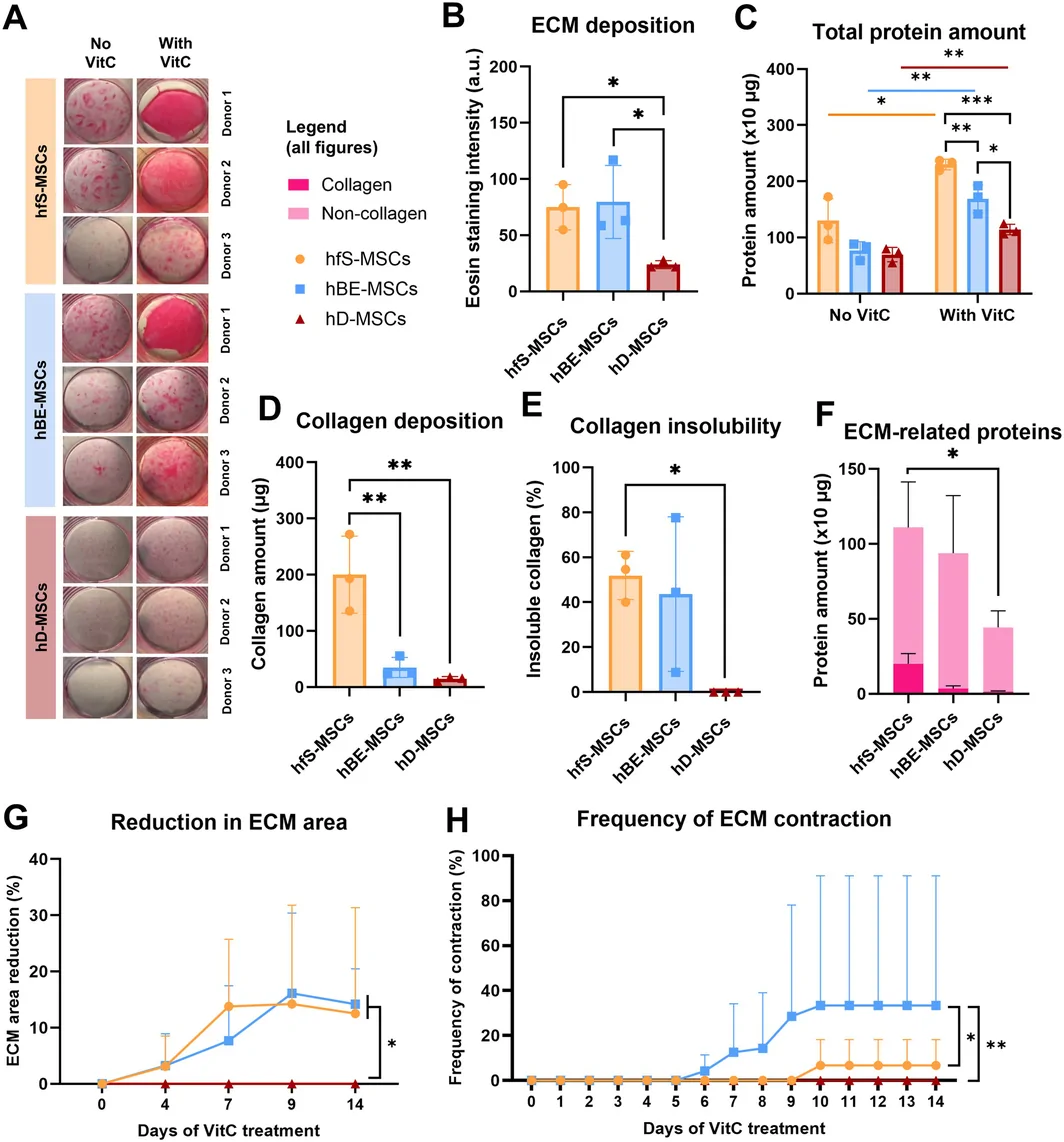
Differential protein expression in hfS‐MSCs, hD‐MSCs and hBE‐MSCs stimulated or not with VITC. One point/donor, donors = 3–4/MSC type. 10 μg/mL VITC concentration used. (A) Wells stained with eosin at Day 9 before any contraction. (B) ECM deposition measured by maximal eosin staining intensity after 14 days with VITC. (C) Maximal protein amount after14 days measured by BCA assay with or without VITC. (D) Maximal collagen amount after 14 days by SIRCOL assay with VITC. (E) Percentage of insoluble collagen at Day 14. (F) ECM‐related proteins: Collagen (soluble + insoluble), in dark pink and non‐collagen content, in light pink; calculated by the difference in total protein between ECM production conditions (10 μg/mL VITC) and standard conditions (0 μg/mL VITC), with insoluble collagen added, as it is not detected by the BCA assay: $\mathrm{ECM}\;\text{related proteins}={\text{Total protein}}_{\mathrm{ECM}\;\text{production conditions}}-{\text{Total protein}}_{\text{standard conditions}}+\text{insoluble collagen}$. (B–F) 1 well/donor (two technical replicates). MSC type comparisons were performed using one‐way ANOVA with Tukey's post hoc test. *p < 0.05, **p < 0.01, ***p < 0.001. (G) Percentage of ECM area reduction from Days 0 to 14. One point is the average of three donors, 1 well/donor. (H) Frequency of contraction—percentage of wells where contraction (~100% ECM area reduction)—occurred from Days 0 to 14. One point is the average of three donors, 10 wells/donor. (G, H) MSC type comparisons across all time points were performed using two‐way ANOVA with Tukey's post hoc test. The hD‐MSCs group, which exhibited no variance due to all values being zero, was included; however, results involving this group should be interpreted with caution. *p ≤ 0.05, **p < 0.01.

hfS‐MSCs produced significantly more collagen, as measured by SIRCOL assay, than hBE‐MSCs and hD‐MSCs, 6‐ and 14‐fold, respectively (Figure 4D). At Day 14, hfS‐MSCs showed significantly higher insoluble collagen levels compared to hD‐MSCs (Figure 4E). Although hBE‐MSCs showed insoluble collagen levels comparable to hfS‐MSCs and all hBE‐MSC biological replicates displayed higher values than hD‐MSCs, this difference did not reach statistical significance, likely due to high inter‐donor variability within the hBE‐MSC group (Figure 4E).

Consistent with eosin staining, the calculated amounts of ECM‐related proteins from SIRCOL and BCA assays showed that hfS‐MSCs secreted significantly more total ECM proteins than hD‐MSCs (Figure 4F). hBE‐MSCs also displayed higher protein levels compared with hD‐MSCs, although this difference was not statistically significant, despite all values being consistently higher (Figure 4F). Additionally, we observed differences in the proportion of collagenous (collagen proteins) versus non‐collagenous proteins (proteoglycans, glycoproteins, ECM‐affiliated proteins, ECM regulators, secreted factors [28]) between MSC populations (Figure 4F, Table 2). hfS‐MSCs secreted ECM proteins in which 20% were collagens and 80% were non‐collagenous proteins, whereas hBE‐MSCs and hD‐MSCs secreted ECM proteins in which the majority were non‐collagenous (96%–97%) and only 3%–4% were collagens (Table 2).

**TABLE 2:** Proportion of collagenous versus non‐collagenous proteins in the ECM.

Cells	Total ECM‐related proteins							
	Collagen (%)				Non‐collagenous proteins (%)			
	Donor 1	Donor 2	Donor 3	Mean	Donor 1	Donor 2	Donor 3	Mean
hfS‐MSCs	32.5	15.7	10.7	19.7	67.5	84.3	89.3	80.4
hBE‐MSCs	4.0	3.0	4.0	3.7	95.9	97.0	96.0	96.3
hD‐MSCs	3.1	3.2	3.5	3.3	96.9	96.9	96.5	96.7

#### 3.3.2 ECM Contraction

Both the area of ECMs derived from hfS‐MSCs and hBE‐MSCs suffered a slight ~13% contraction and no contraction was observed in hD‐MSC‐derived ECM (Figure 4G). However, hBE‐MSC‐derived ECM exhibited significantly increased frequency of ECM contraction compared to ECMs derived from hfS‐MSCs and hD‐MSCs (Figure 4H).

#### 3.3.3 Gene Expression

We examined the differential expression of key ECM and scarring‐associated genes—major collagen types, highly expressed glycoproteins and proteoglycans, ECM regulators and a myofibroblast marker—at 48 h across MSC populations in the presence and absence of VITC.

Under ECM‐producing conditions with VITC supplementation, hfS‐MSCs exhibited significantly higher COL1A1 expression than hD‐MSCs (Figure 5A). Although COL1A1 expression was consistently higher in hfS‐MSCs under standard medium conditions, this difference did not reach statistical significance, likely due to substantial variability among biological replicates (Figure 5A). No statistically significant differences in COL3A1 expression were detected among MSC populations; however, hfS‐MSCs displayed a tendency towards higher expression levels (Figure 5B). COL5A1 expression was significantly elevated in hfS‐MSCs under both standard and ECM‐producing conditions compared with the other MSC populations (Figure 5C).

**FIGURE 5:**
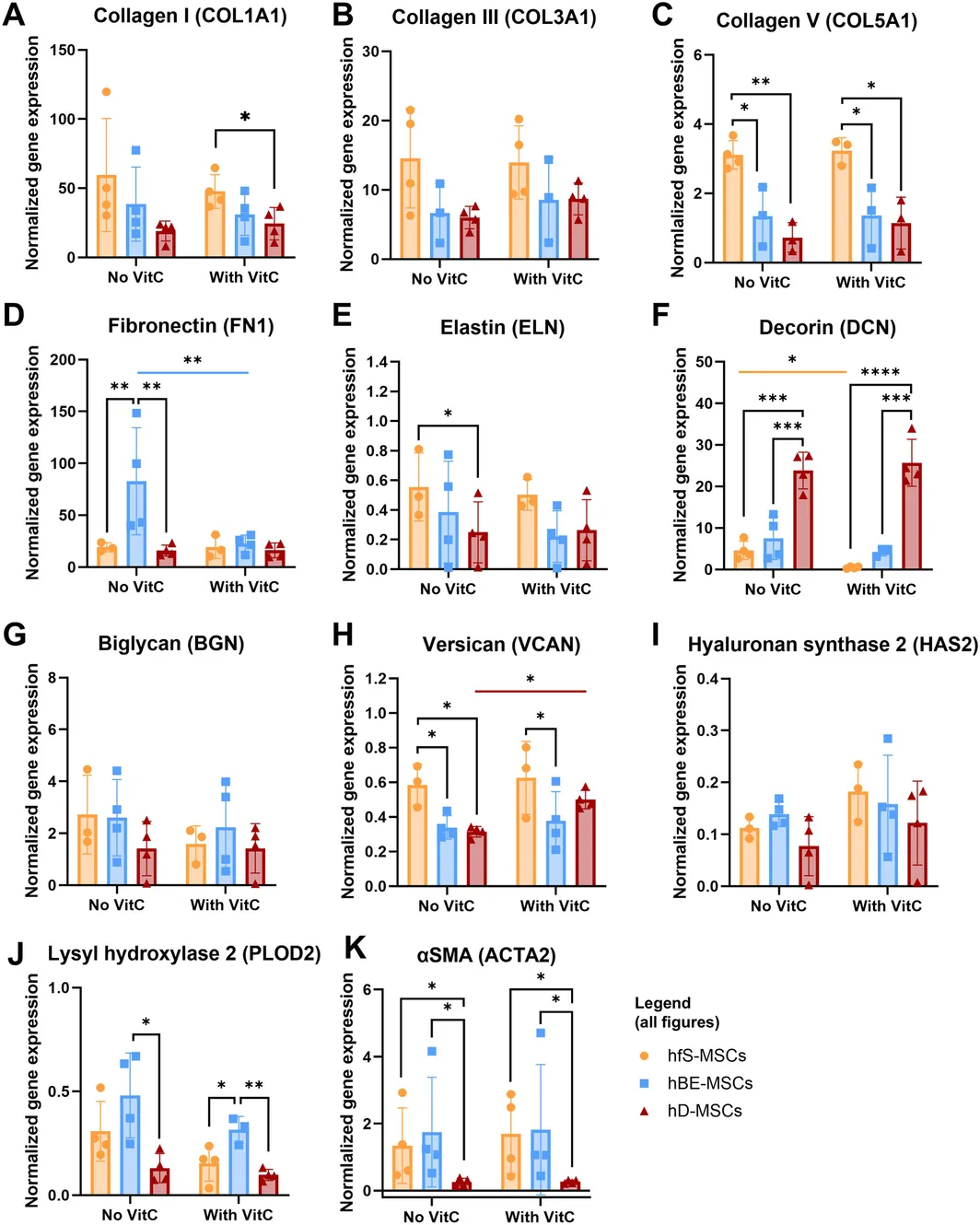
Differential gene expression in hfS‐MSCs, hBE‐MSCs and hD‐MSCs stimulated or not with VITC. One point/donor, donors = 3–4/MSC type. 10 μg/mL VITC concentration used. Normalised ECM or wound‐related gene expression at 48 h: (A–C) Type I, III and V collagens; (D, E) glycoproteins fibronectin and elastin; (F–H) proteoglycans decorin, biglycan, versican; (I, J) ECM regulators hyaluronan synthase 2 and lysyl hydroxylase 2; (K) myofibroblast marker α‐SMA. (A–K) 1 well/donor (two technical replicates). Comparisons among MSC types were performed using one‐way ANOVA with Tukey's post hoc test or Kruskal–Wallis test with Dunn's post hoc test, depending on data normality. For each MSC type, groups with and without VITC were compared using the same statistical approach. *p < 0.05, **p < 0.01, ***p < 0.001.

Both FN1 and ELN exhibited significant differences among MSC populations exclusively under standard medium conditions. FN1 expression was increased in hBE‐MSCs compared to the other MSC populations, whereas ELN expression was significantly higher in hfS‐MSCs than in hD‐MSCs (Figure 5D,E).

hD‐MSCs presented significantly higher DCN expression than other MSC populations, independently of conditions (Figure 5F). In contrast, VCAN expression was significantly higher in hfS‐MSCs under standard medium conditions versus both hBE‐MSCs and hD‐MSCs and this difference persisted under ECM conditions when compared to hBE‐MSCs (Figure 5H). BGN and HAS2 did not show significant differences between MSC populations (Figure 5G–I). PLOD2 expression was significantly higher in hBE‐MSCs than in hD‐MSCs under standard medium conditions; moreover, under ECM‐producing conditions, hBE‐MSCs exhibited significantly higher expression than the other MSC populations (Figure 5J). Finally, ACTA2 was significantly increased in hfS‐MSCs and hBE‐MSCs versus hD‐MSCs, independently of culture conditions (Figure 5K).

Significant differences were also observed between standard and ECM‐producing conditions. VITC reduced DCN expression 10‐fold in hfS‐MSCs (Figure 5F), decreased FN1 in hBE‐MSCs (Figure 5D) and increased VCAN in hD‐MSCs (Figure 5H).

The collagen I:III ratio differs between healthy skin and scar tissue as a result of scarring, while the collagen I:V ratio regulates fibril amount and morphology, impacting wound healing [29, 30, 31, 32]. hBE‐MSCs and hD‐MSCs showed a 1.5‐fold higher COL1A1/COL5A1 expression ratio than hfS‐MSCs under ECM production conditions, with a similar increase in hD‐MSCs compared to hfS‐MSCs under standard conditions (Table 3). Additionally, hBE‐MSCs presented increased COL1A1/COL3A1 ratio versus hD‐MSCs under standard conditions (Table 3).

**TABLE 3:** Average of gene expression ratios of Types I:III and I:V collagen based on the results presented in Figure 5.

Cells	No VITC		With VITC	
	I:III	I:V	I:III	I:V
hfS‐MSCs	4.09	19.2^b^	3.41	14.8^a^, ^b^
hBE‐MSCs	5.79^a^	28.8	3.63	22.9
hD‐MSCs	3.17	26.3	2.81	21.5

^a^ Fold changes > 1.5 compared to hD‐MSCs.

^b^ Fold changes > 1.5 compared to hBE‐MSCs.

### 3.4 Differences Between Foetal and Adult Skin

Herovici staining was performed on foetal and adult skin sections to assess collagen maturity (Figure 6A, representative images). A significant interaction between skin origin (foetal or adult) and collagen maturity (*p ≤* 0.0001; Figure 6B) indicated that the relative proportion of mature versus immature collagen differed between foetal and adult skin. Foetal skin contained a significantly higher proportion of immature collagen than adult skin, while adult skin contained a significantly higher proportion of mature collagen than foetal skin (Figure 6B).

**FIGURE 6:**
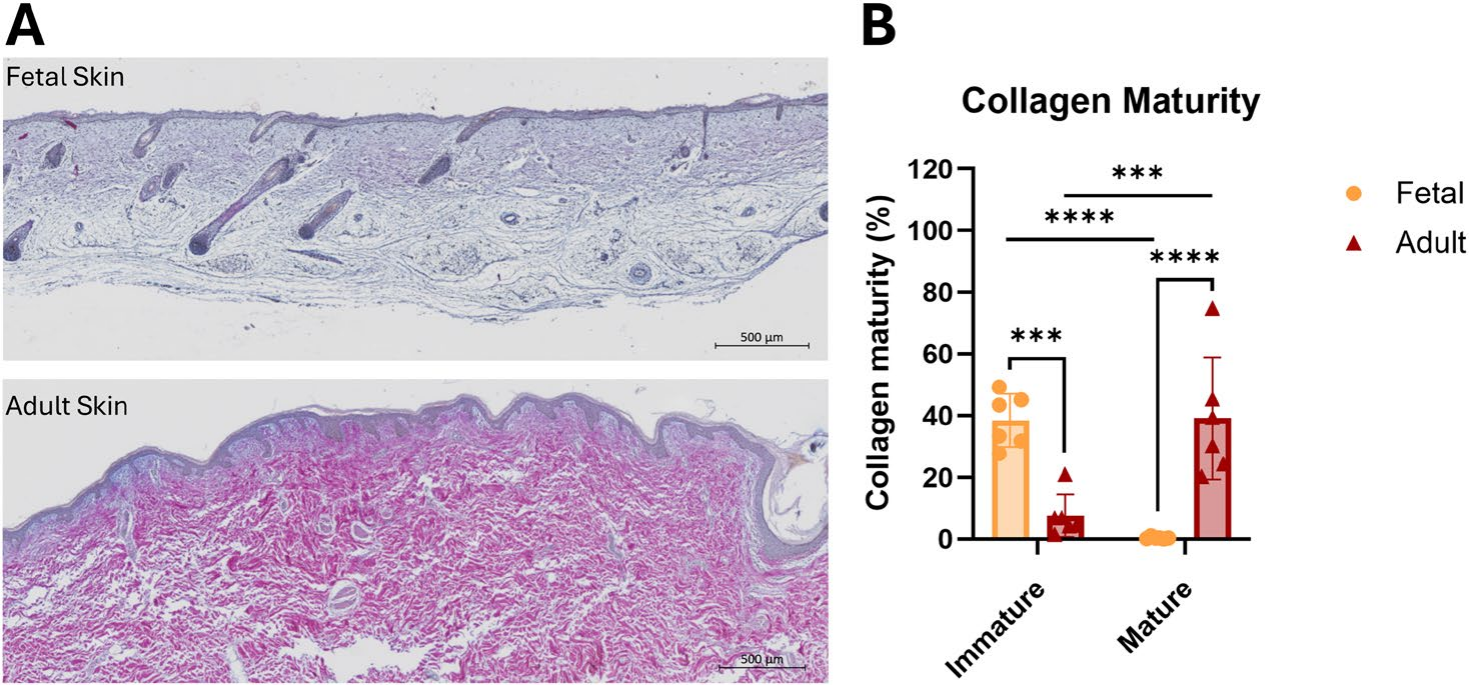
Collagen maturity differences between foetal and adult skin assessed by Herovici staining. One point/donor, donors = 6/tissue type. (A) Representative Herovici‐stained sections of foetal and adult skin; mature collagen stains pink/red, immature collagen stains blue (scale bar = 500 μm). (B) Percentage of mature and immature collagen relative to total tissue area, foetal versus adult. Comparisons were performed using a two‐way ANOVA (origin × maturity) with uncorrected Fisher's least significant difference as post hoc test. ***p < 0.001, ****p < 0.0001.

## 4 Discussion

### 4.1 Characterisation of MSCs

All cell populations met the revised ISCT criteria regarding adherence and surface marker expression [4]. CD105 expression was moderate, with high positivity (92%) but relatively low MFI, consistent with previous reports [33, 34]. CD34 is no longer considered definitive for MSC identification [4], as it is expressed by embryonic fibroblasts/stem cells, adult dermal and eschar MSCs [20, 33, 35]. CD34 expression in MSCs is associated with enhanced colony formation, proliferation and endothelial transdifferentiation potential [20]. All populations expressed CD34, with the highest levels in hfS‐MSCs, suggesting greater potential for these functions.

### 4.2 Optimisation of ECM Production

We optimised culture conditions for ECM production in three MSC populations. hD‐MSCs did not produce ECM in CnT‐PR‐ECM, so this medium was excluded. VITC supplementation was necessary for collagen and ECM production, as it acts as a cofactor for collagen synthesis and crosslinking enzymes [27, 36]. Vitamin C (or derivatives) induces collagen and glycosaminoglycan deposition in skin fibroblasts [15, 17, 18] and increases fibroblast proliferation [37].

hD‐MSCs showed increased COL1A1 expression 48 h post‐VITC, whereas hBE‐MSCs and hfS‐MSCs may exhibit an earlier transcriptional peak associated with rapid ECM deposition. High VITC concentrations (180 μg/mL) reduced COL1A1 expression in hBE‐MSCs and hfS‐MSCs, potentially due to oxidative stress [38, 39], although collagen protein levels were unaffected. Higher VITC doses increased ECM contraction, consistent with previous reports [16], but did not alter ACTA2 expression. Overall, 10 μg/mL VITC provided optimal ECM production while minimising contraction.

### 4.3 ECM Differences Across MSC Populations and Their Tissues of Origin

Previous studies on skin cell‐derived ECM have primarily compared neonatal/adult and foetal fibroblasts under standard culture conditions, which fail to mimic dynamic ECM production during wound healing [3]. Furthermore, ECM differences between hD‐MSCs, hfS‐MSCs and hBE‐MSCs remained unexplored. Here, we compared ECM deposition and related gene expression in primary human MSCs from tissues associated with regeneration (foetal skin) or repair (adult skin and burn eschar) under standard and ECM‐producing conditions.

Overall, hfS‐MSCs and hBE‐MSCs produced more ECM than hD‐MSCs, indicating higher baseline ECM production. In hfS‐MSCs, this may reflect a constitutive regenerative phenotype, with elevated ECM production present at baseline rather than induced only after injury, as occurs in adult cells. This potentially contributes to accelerated foetal wound healing. However, high ECM production alone did not define a regenerative phenotype, as hBE‐MSCs also produced abundant ECM but with a distinct composition.

hfS‐MSC‐derived ECM was markedly collagen‐enriched at both gene and protein levels, consistent with previous observations of accelerated collagen deposition in foetal wounds [3]. This collagen enrichment was associated with higher insoluble collagen fractions, generally indicative of greater collagen crosslinking and fibrillar assembly [40, 41]. hBE‐MSC–derived ECM also showed higher insolubility, although with substantial donor‐to‐donor variability and without collagen enrichment, suggesting a distinct phenotype, capable of high‐volume ECM production without the collagen‐specific signature characteristic of hfS‐MSCs.

Interestingly, although hfS‐MSCs produced abundant insoluble collagen in vitro, our tissue analysis showed that foetal skin contained a higher proportion of immature collagen than adult skin. This suggests that foetal collagen can rapidly form an insoluble matrix without progressing to the mature fibrillar organisation typical of adult skin. We believe this may occur by ongoing matrix turnover that favours persistence of immature collagen over accumulation of mature fibres, though direct evidence would be needed to confirm this.

Collagen composition further distinguished MSC‐derived ECM between populations. hfS‐MSCs showed higher collagen I and V expression compared with hD‐MSCs, aligning with previous research in fibroblasts. The decreased collagen I:V gene expression ratio in hfS‐MSCs, together with the role of collagen V in supporting wound healing [3], suggests that increased collagen V proportion may contribute to an enhanced healing trajectory. Conversely, hBE‐MSCs showed an increased collagen I:III gene expression ratio without any ECM‐production stimulation, suggesting an intrinsic tendency towards lower Type III collagen production. This altered composition may favour thicker collagen fibres, decreased collagen turnover rate and myofibroblast differentiation, features associated with scar formation [3, 42]. However, these interpretations are based on transcriptional data and require validation at the protein and structural levels.

Other ECM components further supported these distinct matrix profiles. hBE‐MSCs secreted a higher proportion of non‐collagenous components, with markedly increased fibronectin expression in the absence of VITC, potentially reflecting an early‐stage matrix profile. In contrast, elevated versican expression in hfS‐MSCs, particularly compared to hBE‐MSCs under ECM‐producing conditions, may contribute to accelerated wound healing via enhanced fibroblast and keratinocyte proliferation, migration and ECM synthesis [3, 43, 44]. Higher elastin expression in unstimulated hfS‐MSCs likely reflects its developmental role in ECM formation. Decorin expression was reduced in hfS‐MSCs and hBE‐MSCs compared with hD‐MSCs, particularly, under ECM‐producing conditions, consistent with previous findings in foetal rat fibroblasts [3]. As decorin regulates collagen fibril organisation and inflammation [10, 45, 46], its reduced expression may contribute to altered matrix architecture and inflammation resolution. Biglycan expression was similar between MSC populations, consistent with rat models [3].

PLOD2 (ECM regulator) marker, associated with collagen crosslinking [47], further differentiated hBE‐MSCs. Increased PLOD2 expression compared with healthy skin MSCs may indicate lysine overhydroxylation and pyridinoline crosslink formation, leading to fibrotic collagen accumulation [48]. Although protein‐level validation and LH2 activity analysis are required, increased PLOD2 supports a scar‐associated ECM profile. In animal studies, α‐SMA expression is absent in early foetal wounds [3], indicating absence of myofibroblasts, distinct from what we observe in vitro. Although ACTA2 expression was increased in both BE‐MSCs and hfS‐MSCs, only hBE‐MSC‐derived ECM exhibited higher contraction compared with healthy skin MSCs, supporting a more contractile and scar‐associated phenotype.

Together, these findings demonstrate distinct ECM programmes across MSC populations and tissue origins. Foetal skin‐derived MSCs generated more rapidly a highly insoluble collagen‐enriched matrix, associated with decreased collagen I:V ratio and increased versican gene expression. In vivo, foetal skin has a markedly higher proportion of immature collagen. Conversely, hBE‐MSCs produce similarly abundant ECM but with increased non‐collagenous components, enhanced contraction, together with increased PLOD2, collagen I:III ratio and baseline fibronectin gene expression. These distinct ECM programmes identify potential biomarkers distinguishing regenerative from fibrotic healing and may guide future strategies to improve burn wound repair.

### 4.4 Limitations and Future Perspectives

A limitation of our study is that MSC characterisation was performed on a separate donor batch, although at least one donor from each MSC population used in ECM experiments was characterised by flow cytometry. All cells were isolated using the same protocol and showed consistent marker expression across donors and alignment with previous findings [33], supporting the reproducibility of our approach. Previous studies from our laboratory contributed additional characterisation of these MSC populations, demonstrating that hBE‐MSCs have trilineage potential, while hD‐MSCs exhibited chondrogenic potential only [33], potentially reflecting enrichment of fibroblast‐like cells within the MSC spectrum. Future studies using larger donor cohorts, self‐renewal capacity assessment and single‐cell or multi‐omics approaches will help define MSC heterogeneity and identify functional subpopulations, including fibroblastic, myofibroblastic and progenitor/stem cells across MSC populations.

A further limitation is that VITC‐induced ECM production represents an integrated response involving collagen maturation, matrix assembly, proliferation, redox regulation and gene expression changes [27, 36, 37, 38, 39]. Since ECM production was not normalised to cell number, we cannot distinguish increased ECM production per cell from increased numbers of ECM‐producing cells. Future studies incorporating cell density normalisation, proliferation assays, deeper gene expression profiling and redox analyses would clarify the relative contributions of these factors.

The relatively small donor cohort (*n* = 3–4 per group), particularly for heterogeneous tissues such as burn eschar, may have limited detection of subtle differences between MSC populations. The high variability in donor age (9–76 years) may have further increased biological heterogeneity, as aging is associated with reduced fibroblast activity and impaired collagen synthesis [49]. Additionally, the range of post‐burn day (PBD) (11–30 days) encompassed overlapping inflammatory, proliferative and early remodelling phases [50, 51], which may contribute to donor‐dependent differences in ECM production. The uneven source of MSCs amplified this limitation: adult skin samples were primarily obtained from the abdominal region, whereas the anatomical origin of burn eschar and foetal skin was unknown. Since MSCs retain positional memory, this may introduce heterogeneity in cell behaviour, including proliferation, ECM‐related gene expression and matrisome secretion profiles [52, 53]. Furthermore, hD‐MSCs and hBE‐MSCs were predominantly from female donors in the ECM production studies, while the sex of certain foetal donors was unknown. Since sex hormones influence wound healing (oestrogen promotes healing, while androgens inhibit it) [54], this sex bias may limit the generalizability of our findings to male adult donors and the unknown foetal sex further constrains the translational interpretation of results. Future studies using larger, sex‐balanced cohorts and controlling for anatomical origin, age and post‐burn stage will help define their individual contributions to ECM production and scar formation.

Although this study characterised ECM production, composition and contraction using complementary approaches, future studies could expand both the mechanistic understanding and the range of ECM‐ or wound‐related targets assessed. MSCs and their derived matrices could be further profiled using multi‐omics strategies to reveal additional regulators or matrix components not examined here and validated in vitro at the protein level over time and in vivo at the tissue level. Functional and structural ECM properties could be further explored using biomechanical testing, decellularized matrix analyses or ultrastructural imaging. Finally, the contribution of specific ECM components could be assessed by selective addition or inhibition in culture systems or engineered scaffolds. Together, these approaches would provide a more comprehensive understanding of ECM regulation and its translational potential for regenerative therapies.

## Author Contributions

M.L.N.P.G., P.A.J.K., E.M., H.W.M.N. and B.K.H.L.B. conceptualized the project, which was administered by B.K.H.L.B. M.L.N.P.G. developed the methodology, generated thevisualizations, wrote the original manuscript draft, and performed the coreinvestigation and formal analysis. M.V. conducted the flow cytometry andhistological experiments and analyses, while A.D. performed the gene expressionexperiments and analyses under the supervision of M.L.N.P.G. E.M. wasresponsible for funding acquisition. Finally, P.A.J.K., H.W.M.N. and B.K.H.L.B.provided overall supervision for M.L.N.P.G., and all authors contributed to thereview and editing of the manuscript.

## Funding

This work was supported by Horizon 2020 Framework Programme (955722).

## Conflicts of Interest

The authors declare no conflicts of interest.

## Supporting information


**Figure S1:** Gating strategy for the characterisation of MSCs.
**Table S1:** Panels of antibodies used in flow cytometry to characterise MSCs.
**Table S2:** Sequences of primer pairs (5′–3′).
**Figure S2:** Macroscopic observation of ECM produced in vitro after 21 days of culture.
**Figure S3:** Maximal ratio of insoluble/soluble collagen amounts detected using the SIRCOL collagen assays over the 14 days of VITC treatment (10, 65 and 180 μg/mL).
**Figure S4:** MSC populations and their ECM stained with eosin at Days 0, 4, 7, 9 and 14 across VITC concentrations (0—control, 10, 65 and 180 μg/mL).

## Data Availability

The data that supports the findings of this study are available in the Supporting Information of this article.

## References

[1] W. Żwierełło , K. Piorun , M. Skórka‐Majewicz , A. Maruszewska , J. Antoniewski , and I. Gutowska , “Burns: Classification, Pathophysiology, and Treatment: A Review,” International Journal of Molecular Sciences 24 (2023): 3749. 10.3390/ijms24043749 PMC9959609 36835171

[2] M. G. Jeschke , M. E. van Baar , M. A. Choudhry , K. K. Chung , N. S. Gibran , and S. Logsetty , “Burn Injury,” Nature Reviews Disease Primers 6 (2020): 11. 10.1038/s41572-020-0145-5 PMC7224101 32054846

[3] M. Gomes , P. A. J. Krijnen , E. Middelkoop , H. W. M. Niessen , and B. Boekema , “Fetal Skin Wound Healing: Key Extracellular Matrix Components and Regulators in Scarless Healing,” Journal of Investigative Dermatology 145 (2025): 280–302. 10.1016/j.jid.2024.05.027 39152955

[4] S. Viswanathan , Y. Shi , J. Galipeau , et al., “Mesenchymal Stem Versus Stromal Cells: International Society for Cell & Gene Therapy (Isct) Mesenchymal Stromal Cell Committee Position Statement on Nomenclature,” Cytotherapy 21 (2019): 1019–1024. 10.1016/j.jcyt.2019.08.002 31526643

[5] I. A. Darby and A. Desmoulière , “Scar Formation: Cellular Mechanisms,” in Textbook on Scar Management: State of the Art Management and Emerging Technologies, ed. L. Téot , T. A. Mustoe , E. Middelkoop , and G. G. Gauglitz (Springer, 2020), 19–26. 36351123

[6] R. B. Diller and A. J. Tabor , “The Role of the Extracellular Matrix (ECM) in Wound Healing: A Review,” Biomimetics 7 (2022): 7. 10.3390/biomimetics7030087 PMC9326521 35892357

[7] J. W. Shin , S. H. Kwon , J. Y. Choi , et al., “Molecular Mechanisms of Dermal Aging and Antiaging Approaches,” International Journal of Molecular Sciences 20 (2019): 2126. 10.3390/ijms20092126 PMC6540032 31036793

[8] M. M. Smith and J. Melrose , “Proteoglycans in Normal and Healing Skin,” Advances in Wound Care 4 (2015): 152–173. 10.1089/wound.2013.0464 PMC4352701 25785238

[9] M. G. Kinsella , S. L. Bressler , and T. N. Wight , “The Regulated Synthesis of Versican, Decorin, and Biglycan: Extracellular Matrix Proteoglycans That Influence Cellular Phenotype,” Critical Reviews in Eukaryotic Gene Expression 14 (2004): 203–234. 10.1615/critreveukaryotgeneexpr.v14.i3.40 15248816

[10] Y. Li , Y. Liu , W. Xia , D. Lei , J. J. Voorhees , and G. J. Fisher , “Age‐Dependent Alterations of Decorin Glycosaminoglycans in Human Skin,” Scientific Reports 3 (2013): 2422. 10.1038/srep02422 PMC3741628 23939413

[11] L. Nabai , A. Pourghadiri , and A. Ghahary , “Hypertrophic Scarring: Current Knowledge of Predisposing Factors, Cellular and Molecular Mechanisms,” Journal of Burn Care & Research 41 (2020): 48–56. 10.1093/jbcr/irz158 PMC6990447 31999336

[12] F. Grinnell , “Fibronectin and Wound Healing,” Journal of Cellular Biochemistry 26 (1984): 107–116. 10.1002/jcb.240260206 6084665

[13] M. Terajima , Y. Taga , T. Nakamura , et al., “Lysyl Hydroxylase 2 Mediated Collagen Post‐Translational Modifications and Functional Outcomes,” Scientific Reports 12 (2022): 14256. 10.1038/s41598-022-18165-0 PMC9395344 35995931

[14] A. J. van der Slot , A. M. Zuurmond , A. F. Bardoel , et al., “Identification of plod2 as Telopeptide Lysyl Hydroxylase, an Important Enzyme in Fibrosis,” Journal of Biological Chemistry 278 (2003): 40967–40972. 10.1074/jbc.M307380200 12881513

[15] R. Hata and H. Senoo , “L‐Ascorbic Acid 2‐Phosphate Stimulates Collagen Accumulation, Cell Proliferation, and Formation of a Three‐Dimensional Tissuelike Substance by Skin Fibroblasts,” Journal of Cellular Physiology 138 (1989): 8–16. 10.1002/jcp.1041380103 2910890

[16] N. Boyera , I. Galey , and B. A. Bernard , “Effect of Vitamin c and Its Derivatives on Collagen Synthesis and Cross‐Linking by Normal Human Fibroblasts,” International Journal of Cosmetic Science 20 (1998): 151–158. 10.1046/j.1467-2494.1998.171747.x 18505499

[17] Y. Kishimoto , N. Saito , K. Kurita , K. Shimokado , N. Maruyama , and A. Ishigami , “Ascorbic Acid Enhances the Expression of Type 1 and Type 4 Collagen and svct2 in Cultured Human Skin Fibroblasts,” Biochemical and Biophysical Research Communications 430 (2013): 579–584. 10.1016/j.bbrc.2012.11.110 23228664

[18] J. C. Geesin , D. Darr , R. Kaufman , S. Murad , and S. R. Pinnell , “Ascorbic Acid Specifically Increases Type i and Type Iii Procollagen Messenger Rna Levels in Human Skin Fibroblast,” Journal of Investigative Dermatology 90 (1988): 420–424. 10.1111/1523-1747.ep12460849 3351329

[19] H. Green and B. Goldberg , “Collagen Synthesis by Human Fibroblast Strains,” Proceedings of the Society for Experimental Biology and Medicine 117 (1964): 258–261. 10.3181/00379727-117-29551 14223286

[20] L. E. Sidney , M. J. Branch , S. E. Dunphy , H. S. Dua , and A. Hopkinson , “Concise Review: Evidence for cd34 as a Common Marker for Diverse Progenitors,” Stem Cells 32 (2014): 1380–1389. 10.1002/stem.1661 PMC4260088 24497003

[21] A. H. Fischer , K. A. Jacobson , J. Rose , and R. Zeller , “Hematoxylin and Eosin Staining of Tissue and Cell Sections,” CSH Protocols 2008 (2008): pdb.prot4986. 10.1101/pdb.prot4986 21356829

[22] C. Herovici , “Picropolychrome: Histological Staining Technic Intended for the Study of Normal and Pathological Connective Tissue,” Revue Française d'Études Cliniques et Biologiques 8 (1963): 88–89. 13954039

[23] M. E. H. Jaspers , F. Feroldi , M. Vlig , J. F. de Boer , and P. P. M. van Zuijlen , “In Vivo Polarization‐Sensitive Optical Coherence Tomography of Human Burn Scars: Birefringence Quantification and Correspondence With Histologically Determined Collagen Density,” Journal of Biomedical Optics 22 (2017): 1–8. 10.1117/1.JBO.22.12.121712 29264892

[24] The R Foundation , “The r Project for Statistical Computing,” 2026, https://www.r‐project.org/.

[25] H. Wickham and J. Bryan , “Readxl: Read Excel Files,” 2025, 10.32614/CRAN.package.readxl.

[26] H. Wickham , M. Averick , J. Bryan , et al., “Welcome to the Tidyverse,” Journal of Open Source Software 4 (2919): 1686.

[27] B. Piersma , O. Y. Wouters , S. de Rond , M. Boersema , R. A. F. Gjaltema , and R. A. Bank , “Ascorbic Acid Promotes a tgfβ1‐Induced Myofibroblast Phenotype Switch,” Physiological Reports 5 (2017): e13324. 10.14814/phy2.13324 PMC5599854 28904079

[28] X. Shao , C. D. Gomez , N. Kapoor , et al., “Matrisomedb 2.0: 2023 Updates to the Ecm‐Protein Knowledge Database,” Nucleic Acids Research 51 (2023): D1519–D1530. 10.1093/nar/gkac1009 PMC9825471 36399478

[29] M. Zhou , M. P. Gomes , A. Elgersma , H. I. Korkmaz , B. Boekema , and M. L. Groot , “Multiscale Investigation of Collagen Structure in Human Skin and Gel Matrices Using Polarization Resolved Second Harmonic Generation Microscopy,” Scientific Reports 15 (2025): 20025. 10.1038/s41598-025-02536-4 PMC12144187 40481016

[30] M. Sun , S. Chen , S. M. Adams , et al., “Collagen v Is a Dominant Regulator of Collagen Fibrillogenesis: Dysfunctional Regulation of Structure and Function in a Corneal‐Stroma‐Specific col5a1‐Null Mouse Model,” Journal of Cell Science 124 (2011): 4096–4105. 10.1242/jcs.091363 PMC3244988 22159420

[31] C. Y. Su , T. Y. Liu , H. V. Wang , and W. C. Yang , “Histopathological Study on Collagen in Full‐Thickness Wound Healing in Fraser's Dolphins ( *lagenodelphis hosei* ),” Animals 13 (2023): 13. 10.3390/ani13101681 PMC10215821 37238111

[32] W. K. Stadelmann , A. G. Digenis , and G. R. Tobin , “Physiology and Healing Dynamics of Chronic Cutaneous Wounds,” American Journal of Surgery 176 (1998): 26s–38s. 10.1016/s0002-9610(98)00183-4 9777970

[33] V. C. van der Veen , M. Vlig , F. J. van Milligen , S. I. de Vries , E. Middelkoop , and M. M. Ulrich , “Stem Cells in Burn Eschar,” Cell Transplantation 21 (2012): 933–942. 10.3727/096368911X600993 21944933

[34] M. Orciani , M. A. Mariggiò , C. Morabito , G. Di Benedetto , and R. Di Primio , “Functional Characterization of Calcium‐Signaling Pathways of Human Skin‐Derived Mesenchymal Stem Cells,” Skin Pharmacology and Physiology 23 (2010): 124–132. 10.1159/000270383 20051713

[35] B. Nazari , L. M. Rice , G. Stifano , et al., “Altered Dermal Fibroblasts in Systemic Sclerosis Display Podoplanin and cd90,” American Journal of Pathology 186 (2016): 2650–2664. 10.1016/j.ajpath.2016.06.020 PMC5222985 27565038

[36] T. Chaitrakoonthong , R. Ampornaramveth , and P. Kamolratanakul , “Rinsing With l‐Ascorbic Acid Exhibits Concentration‐Dependent Effects on Human Gingival Fibroblast In Vitro Wound Healing Behavior,” International Journal of Dentistry 2020 (2020): 4706418. 10.1155/2020/4706418 PMC7115167 32273893

[37] B. M. Mohammed , B. J. Fisher , D. Kraskauskas , et al., “Vitamin c Promotes Wound Healing Through Novel Pleiotropic Mechanisms,” International Wound Journal 13 (2016): 572–584. 10.1111/iwj.12484 PMC7949787 26290474

[38] J. Kaźmierczak‐Barańska , K. Boguszewska , A. Adamus‐Grabicka , and B. T. Karwowski , “Two Faces of Vitamin c‐Antioxidative and Pro‐Oxidative Agent,” Nutrients 12 (2020): 1501. 10.3390/nu12051501 PMC7285147 32455696

[39] Y. Morel and R. Barouki , “Repression of Gene Expression by Oxidative Stress,” Biochemical Journal 342, no. Pt 3 (1999): 481–496. PMC1220487 10477257

[40] B. López , R. Querejeta , A. González , M. Larman , and J. Díez , “Collagen Cross‐Linking but Not Collagen Amount Associates With Elevated Filling Pressures in Hypertensive Patients With Stage c Heart Failure: Potential Role of Lysyl Oxidase,” Hypertension 60 (2012): 677–683. 10.1161/HYPERTENSIONAHA.112.196113 22824984

[41] N. R. Davis , O. M. Risen , and G. A. Pringle , “Stable, Nonreducible Cross‐Links of Mature Collagen,” Biochemistry 14 (1975): 2031–2036. 10.1021/bi00680a034 235976

[42] S. W. Volk , Y. Wang , E. A. Mauldin , K. W. Liechty , and S. L. Adams , “Diminished Type Iii Collagen Promotes Myofibroblast Differentiation and Increases Scar Deposition in Cutaneous Wound Healing,” Cells, Tissues, Organs 194 (2011): 25–37. 10.1159/000322399 PMC3128157 21252470

[43] S. Islam , K. Chuensirikulchai , S. Khummuang , et al., “Accumulation of Versican Facilitates Wound Healing: Implication of Its Initial Adamts‐Cleavage Site,” Matrix Biology 87 (2020): 77–93. 10.1016/j.matbio.2019.10.006 31669737

[44] J. González‐Rico , C. Quílez , V. López , A. Muñoz‐Barrutia , J. L. Jorcano , and D. Velasco , “The Role of Hyaluronic Acid and Versican in the Skin Extracellular Matrix,” Biomecánica 27 (2019): 35–49.

[45] K. G. Danielson , H. Baribault , D. F. Holmes , H. Graham , K. E. Kadler , and R. V. Iozzo , “Targeted Disruption of Decorin Leads to Abnormal Collagen Fibril Morphology and Skin Fragility,” Journal of Cell Biology 136 (1997): 729–743. 10.1083/jcb.136.3.729 PMC2134287 9024701

[46] X. Pang , N. Dong , and Z. Zheng , “Small Leucine‐Rich Proteoglycans in Skin Wound Healing,” Frontiers in Pharmacology 10 (2019): 1649. 10.3389/fphar.2019.01649 PMC6997777 32063855

[47] Y. Qi and R. Xu , “Roles of Plods in Collagen Synthesis and Cancer Progression,” Frontiers in Cell and Developmental Biology 6 (2018): 66. 10.3389/fcell.2018.00066 PMC6031748 30003082

[48] A. J. van der Slot , A. M. Zuurmond , A. J. van den Bogaerdt , et al., “Increased Formation of Pyridinoline Cross‐Links due to Higher Telopeptide Lysyl Hydroxylase Levels Is a General Fibrotic Phenomenon,” Matrix Biology 23 (2004): 251–257. 10.1016/j.matbio.2004.06.001 15296939

[49] K. A. Khalid , A. F. M. Nawi , N. Zulkifli , M. A. Barkat , and H. Hadi , “Aging and Wound Healing of the Skin: A Review of Clinical and Pathophysiological Hallmarks,” Life 12 (2022): 12. 10.3390/life12122142 PMC9784880 36556508

[50] W. S. Siu , H. Ma , and P. C. Leung , “Review on Current Advancements in Facilitation of Burn Wound Healing,” Bioengineering 12 (2025): 12. 10.3390/bioengineering12040428 PMC12024970 40281787

[51] P. P. G. Mulder , M. Vlig , E. Fasse , et al., “Burn‐Injured Skin Is Marked by a Prolonged Local Acute Inflammatory Response of Innate Immune Cells and Pro‐Inflammatory Cytokines,” Frontiers in Immunology 13 (2022): 1034420. 10.3389/fimmu.2022.1034420 PMC9703075 36451819

[52] A. M. Sacco , I. Belviso , V. Romano , et al., “Diversity of Dermal Fibroblasts as Major Determinant of Variability in Cell Reprogramming,” Journal of Cellular and Molecular Medicine 23 (2019): 4256–4268. 10.1111/jcmm.14316 PMC6533477 30980516

[53] T. Kirk , A. Ahmed , and E. Rognoni , “Fibroblast Memory in Development, Homeostasis and Disease,” Cells 10 (2021): 10. 10.3390/cells10112840 PMC8616330 34831065

[54] S. C. Gilliver , J. J. Ashworth , and G. S. Ashcroft , “The Hormonal Regulation of Cutaneous Wound Healing,” Clinics in Dermatology 25 (2007): 56–62. 10.1016/j.clindermatol.2006.09.012 17276202

