# Supplementary material for: Differential Extracellular Matrix Production by Mesenchymal Stromal Cells From Foetal Skin, Adult Skin and Burn Eschar
Source: Wound Repair Regen. 2026 Oct 6;34(5):e70202. doi: 10.1111/wrr.70202 (PMC13642972; doi:10.1111/wrr.70202)
Supplement: Supplementary file 1 [file WRR-34-0-s001.docx]

# Supplementary Materials and Methods

## Isolation of Primary Human MSCs

Split-skin grafts (0.3 mm in thickness) from healthy adult skin were harvested using a dermatome (Aesculap AG & Co. KG, Tuttlingen, BW, Germany). Burn wound eschar tissue, fetal skin and adult split-skin grafts were washed with phosphate-buffered saline (PBS, Gibco, Paisley, SCT, UK). The split-skin graft was incubated with 0.25% (w/v) dispase II (Boehringer, Mannheim, BW, Germany) for 25 min at 37ºC, followed by separation of the epidermis from the dermis using tweezers. Tissues were weighed, cut with sterile scissors and incubated with a dispase II/collagenase A (Roche Diagnostics, Mannheim, BW, Germany) solution [both 0.25% (w/v) in PBS] for 1h (fetal skin) or 2 h (adult skin, eschar tissue) at 37ºC under agitation. Enzymes were inactivated using 1 mM EDTA. The cells were separated from tissue debris in a filter chamber (NPBI, Emmer-Compascuum, DR, The Netherlands) or a series of cell-strainers from 500 µm to 70 µm (PluriSelect, Leipzig, SN, Germany), and centrifuged (300g, 5 min).

**Fetal Donor Sex Identification**

### The sex of the fetal donors was determined retrospectively following the characterization and ECM production studies.

### DNA extraction

After thawing, a sample of cell suspension corresponding to 25,000 cells was collected from each donor. Samples were centrifuged at 300g for 10min, the medium was aspirated and 100 µl of lysis buffer (50 mM Tris-HCl pH 8.5, 100 mM NaCl, 1mM EDTA, 0.5% Tween-20, 0.5% NP-40) with proteinase K (1:10; Thermo Fisher Scientific, Waltham, MA, USA) was added for each sample. Samples were incubated for 2h at 55°C, followed by a 10min incubation at 90°C to ensure proteinase K inactivation.

### PCR

For each sample, 0.5 µl of extracted DNA was added to the master mix (2.5 µl buffer, 0.5 µl 10 mM dNTPs, 0.75 µl 50 mM MgCl_2_, 0.1 µl 5 µ/µl TAQ polymerase) and SRY primers (0.5 µl forward, 0.5 µl reverse). SRY primers were designed using *Primer3* and *SNPCheck* (GeneTools, UK) webtools [1], and previously tested. Gel electrophoresis and UV spectrophotometry (ProXima imaging platform; Isogen Life Science, De Meern, UT, Netherlands) were used to visualize the amplified DNA fragments. Quantitative PCR (qPCR) was subsequently performed for the reference gene B2M. Reactions were prepared using SsoAdvanced™ SYBR® Green Supermix (Bio-Rad Laboratories, Hercules, CA, USA) in a 96-well plate format with primers obtained from Biolegio (Biolegio B.V., Nijmegen, GE, Netherlands). qPCR was performed on a CFX Connect™ Real-Time PCR Detection System (Bio-Rad Laboratories, Hercules, CA, USA). Cycling conditions were set according to the manufacturer’s instructions, with an annealing temperature of 60 °C. A negative (female, Cellosaurus cell line BCWM.1 – CVCL A035, 50 ng/µl) and positive (male, cellosaurus cell line OCI-Ly7 – CVCV 1881, 50 ng/µl) DNA controls, as well as RNA free water as a technical negative control, were used. Two technical replicates were used per DNA sample. Samples positive for SRY were assigned as males, and samples negative for SRY and positive for B2M were assigned as female. Primer pairs are detailed in Supplementary Table 2.

## Characterization of Primary Human MSCs

Compensation was applied to correct for spectral overlap between fluorochromes. Initial gating on FSC-A vs SSC-A was used to exclude debris and cell fragments. Singlets were included by gating FCS-H vs FSC-A, followed by excluding dead cells identified by propidium iodide (PI)-positive events. Marker-positive populations were defined by subtracting the fluorescence intensity of unstained controls from stained samples to establish thresholds for positivity. Each marker was analyzed independently on a per-channel basis. Gating, depicted in Supplementary Fig. 1, and data analysis were performed using FlowLogic software (Inivai Technologies, Melbourne, VIC, Australia). Marker expression was quantified only in live cells as the percentage of marker-positive cells and mean fluorescence intensity (MFI).

**Supplementary Fig. 1: Gating strategy for the characterization of MSCs.**

## Quantification of gene expression

### RNA isolation

Each well was washed twice with PBS (Gibco, Paisley, SCT, UK), followed by the addition of 100 µL of TRI Reagent (Sigma-Aldrich, St. Louis, MO, USA). The cell contents and extracellular matrix were scraped from the well surface. RNA isolation was performed by two chloroform extractions: 10 µl added to the initial sample, followed by 500 µl added to 450 µl RNA-free water and the aqueous phase obtained in the first extraction; RNA was precipitated by incubating for 20 min at RT in 2 µl Glycoblue coprecipitant (Invitrogen, Vilnius, Lithuania), 45 µl 3 M sodium acetate, and 450 µl 2-propanol; and two washes with 75% ethanol were performed before RNA elution in RNA-free water. Isolated RNA was incubated at 55°C for 10min before RNA quality and concentration were measured using Nanodrop 2000 spectrophotometer (Thermo Fisher, Waltham, MA, USA).

### cDNA synthesis

For cDNA synthesis, 0.5 to 1.5 µg RNA, RNA-free water and 0.5 µl random primers (Promega, Leiden, ZH, The Netherlands) were incubated for 10 minutes at 70°C in 27 µl. RT-mix [2.5 µl 10mM dNTPs (Gibco), 2 µl 100 mM DTT, 5 µl 5x first strand buffer (Invitrogen), 0.75 µl 20 U/ul RNase inhibitor (Meridian Bioscience, London, ENG, UK), 1 µl 200 U/ul M-MLV RT (Gibco)] was added, followed by 1 hour incubation at 41°C, and 5 minutes at 95°C.

### RT-qPCR analysis

For qPCR, 1 µl cDNA and 9 µl PCR-mix [5 µl SensiFAST SYBR No-ROX mix (Meridian Bioscience), 0.5 µl of each forward and reverse primers, and RNA-free water] were incubated in the following regime in the LightCycle 480 system (Roche, Basel, BS, Switzerland): 2min at 95°C, and 45 cycles of 5s at 95°C and 25s at 59°C. RNA-free water was used as negative technical control.

### Primer design

Primer design was performed using *NCBI Nucleotide* and *NCBI Primer Blast* to obtain the pairs of primers, and *Oligo Analyzer* to check for secondary structures and primer dimers [2, 3]. Primer validation was conducted by analysis of amplification and melting curves. Data was converted and analysed using LC480 Converter and LinRegPCR softwares [4].

### Selection of reference genes

Reference genes were selected among 11 reference genes based on: 1) highest stability values across 7 samples from independent experiments under conditions similar to this study, 2) average Cq value $\leq$20 to avoid reference genes with low expression. Stability was measured using 3 different methods: NormFinder, BestKeeper, and GeNorm; and the optimal number of reference genes was determined using GeNorm [5-7].

## Total protein and collagen quantification

At days 4, 9, and 14, 110 µl/well of 0.1 mg/ml pepsin (Sigma-Aldrich) in 0.5M acetic acid was added per well. ECM and cell content were collected by scraping and aspirating the suspension. Samples were incubated overnight at 4°C with agitation, then centrifuged at 3000g for 10 min to separate the supernatant (soluble fraction) and pellet (insoluble fraction). The soluble fraction was processed using the Soluble Collagen SIRCOL Assay (Biocolor, Carrickfergus, NIR, UK) and the insoluble fraction using the Insoluble Collagen SIRCOL Assay, following manufacturer’s instructions, with adaptations. Total protein in the soluble fraction was measured using the Pierce™ Bicinchoninic Acid (BCA) Protein Assay (Thermo Scientific, Waltham, MA, USA). For protein content <1000 µg, 100 µl of soluble fraction and 100 µl of fragmentation reagent were used for the Soluble and Insoluble Collagen SIRCOL Assay, respectively. For protein content >1000 µg, 50 µl of soluble and 200 µl of fragmentation reagent were used. The insoluble fraction was solubilized in fragmentation reagent for 2 hours, followed by protein quantification by Pierce™ BCA Protein Assay to ensure sufficient content. Insoluble Collagen SIRCOL Assay proceeded with 100 µl of solubilized sample.

Supplementary Table 1: Panels of antibodies used in flow cytometry to characterize MSCs.

| **Panel** | **Antibody** | **Label** | **Dilution** | **Manufacturer** | **Code** |
| --- | --- | --- | --- | --- | --- |
| 1 | CD90 | APC-Fire750 | 1:250 | Biolegend | 328138 |
|  | CD73 | FITC | 1:100 | Biolegend | 344016 |
| 2 | CD90 | APC-Vio770 | 1:250 | Biolegend | 328138 |
|  | CD105 | FITC | 1:100 | Biolegend | 323204 |
|  | CD34 | APC | 1:100 | Biolegend | 343510 |
| 3 | CD90 | APC-Vio770 | 1:250 | Biolegend | 328138 |
|  | CD14 | VioBlue | 1:250 | Miltenyi Biotec | 130-110-582 |
|  | CD19 | FITC | 1:250 | Miltenyi Biotec | 130-120-031 |
|  | CD31 | APC | 1:250 | Biolegend | 303116 |
|  | CD45 | VioGreen | 1:250 | Miltenyi Biotec | 130-110-776 |

Supplementary Table 2: Sequences of primer pairs (5’- 3’).

| Target | Forward primer | Reverse primer |
| --- | --- | --- |
| αSMA (ACTA2) | CCTCCCTTGAGAAGAGTTACG | ATGCTGTTGTAGGTGGTTTCA |
| Collagen I (COL1A1) | AGGGCGACAGAGGCATAAAG | AGGGAGACCGTTGAGTCCAT |
| Collagen III (COL3A1) | CCTGAAGCTGATGGGGTCAA | AGCCTTGCGTGTTCGATATTC |
| Collagen V (COL5A1) | CCCTGACAAGAAGTCCGAAGG | CGTCCACATAGGAGAGCAGTTT |
| Decorin (DCN) | GTCTTCCTCCTTCCCTTACG | ACTCAATCCCAACTTAGCCA |
| Biglycan (BGN) | GCTCCGCATCCACGACAA | GCTCCAGGTTCAAAGCCACT |
| Versican (VCN) | GCAAGAAAGGAACAGTCGCT | CGGATAGTTGGAAGGTGACG |
| Hyaloronan synthase 2 (HAS2) | TCCTCCTGGGTGGTGTGATT | GTGTGTGACTGCAAACGTCAA |
| Fibronectin (FN1) | CCTCACCAACCTCACTCCAG | AGGGCTATTTCCTCCTGTCTCT |
| Elastin (ELN) | GAGCTTTTGCTGGAATCCCA | GCAGTTTCCCTGTGGTGTAG |
| Lysyl hydroxylase 2 (PLOD2) | GGCAAATGGTCTGGGGGAAA | TTCAGTAGTGCAAATCCCTTCGT |
| Reference (YHWAZ) | CATCTTGGAGGGTCGTCTCA | ACTTTGCTCTCTGCTTGTGAA |
| Reference (GPI) | GACGGCGAAGGAGTGGTTTC | AGGGCAATGGAGAGTCCGAT |
| Sex-determining Region Y (SRY) | GGGATGACTGTACGAAAGCC | acccgattgtcCTACAGCTT |
| Reference (B2M) | CTCCCGCTCTGCACCCTCTGT | TCCCGTCGCCGTAGGCCAAA |

# Supplementary RESULTS

**Supplementary Fig. 2: Macroscopic observation of ECM produced in vitro after 21 days of culture.** **a** hfS-MSC-derived ECM in a transwell removed from the plate, well diameter is 24 mm. **b** hfS-MSC-derived ECM removed from the transwell by cutting its bottom. **c** hBE-MSC-derived ECM contracted. Arrow points to the contracted ECM. **a-c** The ECM is outlined with a white dashed line. 1 of 6 wells represented in the picture.

**Supplementary Fig. 3: Maximal ratio of insoluble/soluble collagen amounts detected using the SIRCOL collagen assays over the 14 days of VITC treatment (10, 65 and 180 µg/ml).** One point/donor, each represented by a unique symbol, donors=3/MSC type, 1 well/donor (3 technical replicates). Mann-Whitney test showed no significant differences.

**Supplementary Fig.4: MSC populations and their ECM stained with eosin at days 0, 4, 7, 9 and 14 across VITC concentrations (0 - control, 10, 65 and 180 µg/ml).** One well for each donor, time point, and VITC concentration is represented; donors=3/MSC type.

# SUPPLEMENTARY References

1. Kõressaar T, Lepamets M, Kaplinski L, Raime K, Andreson R, Remm M. Primer3_masker: Integrating masking of template sequence with primer design software. Bioinformatics. 2018;34:1937–8.

2. National library of medicine: National center for biotechnology information. <https://www.ncbi.nlm.nih.gov/> Accessed 2021-2024.

3. IDT: Oligoanalyzer™: Primer analysis tool. <https://eu.idtdna.com/pages/tools/oligoanalyzer> Accessed 2021-2024.

4. Ramakers C, Ruijter JM, Deprez RH, Moorman AF. Assumption-free analysis of quantitative real-time polymerase chain reaction (pcr) data. Neurosci Lett. 2003;339:62–6.

5. Pfaffl MW, Tichopad A, Prgomet C, Neuvians TP. Determination of stable housekeeping genes, differentially regulated target genes and sample integrity: Bestkeeper--excel-based tool using pair-wise correlations. Biotechnol Lett. 2004;26:509–15.

6. Vandesompele J, De Preter K, Pattyn F, Poppe B, Van Roy N, De Paepe A, et al. Accurate normalization of real-time quantitative rt-pcr data by geometric averaging of multiple internal control genes. Genome Biol. 2002;3:Research0034.

7. Andersen CL, Jensen JL, Ørntoft TF. Normalization of real-time quantitative reverse transcription-pcr data: A model-based variance estimation approach to identify genes suited for normalization, applied to bladder and colon cancer data sets. Cancer Res. 2004;64:5245–50.
